# The growing public health concern of nicotine pouch consumption among Pakistani youth

**DOI:** 10.18332/tid/205164

**Published:** 2025-06-05

**Authors:** Rehan Jan, Maryam Jan

**Affiliations:** 1 Department of Medicine, Federal Medical College, Islamabad, Pakistan; 2 Department of Human Nutrition and Dietetics, Ziauddin University, Karachi, Pakistan

**Keywords:** public health, youth, tobacco control, nicotine pouches, Pakistan

**Dear Editor,**

The increasing availability and promotion of nicotine pouches (NPs) in Pakistan is emerging as a significant public health concern – particularly among adolescents and young adults. Within just five years, Pakistan has become the third-largest market for these products globally, with Velo dominating over 85% of the local market through diverse flavors and strengths aimed at younger users^1^.

Recent findings from a multi-district study reveal how widespread and accessible these products have become. In all four provinces, NPs were sold at 7.9% of surveyed point-of-sale (POS) locations, with greater presence in urban than rural areas. Alarmingly, 70% of these outlets placed the products at a child’s eye level and 20% operated within 200 m of schools – many even positioning the pouches near candy displays^1,2^.

These marketing tactics may lead to normalizing nicotine consumption among youth. University students and schoolchildren – many of whom have never smoked before – may be drawn in by the discreet use, fruity flavors, and the perception that these pouches are less harmful than cigarettes^2^. Nearly 23% of retailers admitted to selling them to minors, reflecting a failure of enforcement and the need for immediate oversight^2^.

Nicotine pouches are not without health risks. Studies show links to elevated blood pressure, oral mucosa irritation, and heightened risk for cardiovascular events such as heart attacks and coronary artery disease^3,4^. Furthermore, regular use at a young age can lead to sustained addiction and potentially act as a gateway to other tobacco products^5^.

Given these concerns, immediate policy action is necessary. Governments need to enact tobacco control measures and enforce age controls, restrict sales around schools and prohibit youth-oriented advertising. Public awareness programs need to promote the addictive quality and health consequences of these products, particularly in countries like Pakistan with poor tobacco control legislation. Furthermore, region-focused studies are highly required to determine effective measures within the context of the WHO FCTC MPOWER strategy.

## Data Availability

Data sharing is not applicable to this article as no new data were created.
